# Small molecule kinase inhibitor LRRK2-IN-1 demonstrates potent activity against colorectal and pancreatic cancer through inhibition of doublecortin-like kinase 1

**DOI:** 10.1186/1476-4598-13-103

**Published:** 2014-05-06

**Authors:** Nathaniel Weygant, Dongfeng Qu, William L Berry, Randal May, Parthasarathy Chandrakesan, Daniel B Owen, Sripathi M Sureban, Naushad Ali, Ralf Janknecht, Courtney W Houchen

**Affiliations:** 1 Department of Medicine, University of Oklahoma Health Sciences Center, Oklahoma City, OK, USA; 2 Department of Veterans Affairs Medical Center, Oklahoma City, OK, USA; 3 Peggy and Charles Stephenson Oklahoma Cancer Center, Oklahoma City, OK, USA; 4 Department of Cell Biology, University of Oklahoma Health Sciences Center, Oklahoma City, OK, USA; 5 COARE Biotechnology, Oklahoma City, OK, USA

**Keywords:** DCLK1, LRRK2-IN-1, Tumor stem cell, Small-molecule inhibitor, Kinase inhibitor

## Abstract

**Background:**

Doublecortin-like kinase 1 (DCLK1) is emerging as a tumor specific stem cell marker in colorectal and pancreatic cancer. Previous *in vitro* and *in vivo* studies have demonstrated the therapeutic effects of inhibiting DCLK1 with small interfering RNA (siRNA) as well as genetically targeting the DCLK1^+^ cell for deletion. However, the effects of inhibiting DCLK1 kinase activity have not been studied directly. Therefore, we assessed the effects of inhibiting DCLK1 kinase activity using the novel small molecule kinase inhibitor, LRRK2-IN-1, which demonstrates significant affinity for DCLK1.

**Results:**

Here we report that LRRK2-IN-1 demonstrates potent anti-cancer activity including inhibition of cancer cell proliferation, migration, and invasion as well as induction of apoptosis and cell cycle arrest. Additionally we found that it regulates stemness, epithelial-mesenchymal transition, and oncogenic targets on the molecular level. Moreover, we show that LRRK2-IN-1 suppresses DCLK1 kinase activity and downstream DCLK1 effector c-MYC, and demonstrate that DCLK1 kinase activity is a significant factor in resistance to LRRK2-IN-1.

**Conclusions:**

Given DCLK1’s tumor stem cell marker status, a strong understanding of its biological role and interactions in gastrointestinal tumors may lead to discoveries that improve patient outcomes. The results of this study suggest that small molecule inhibitors of DCLK1 kinase should be further investigated as they may hold promise as anti-tumor stem cell drugs.

## Background

Small-molecule kinase inhibitors hold significant promise in extending lifespan and improving outcomes for cancer patients. Imatinib (Gleevec®), an inhibitor designed to target the BCR-ABL fusion complex in chronic myelogenous leukemia (CML), was the first successful drug in this category and exemplifies the therapeutic potential of these drugs. With this therapy CML has been transformed from an often-fatal malignancy to a manageable condition with survival rates similar to the disease-free population [1]. In solid tumor cancers, many kinase inhibitors such as sorafenib and gefitinib have been shown to extend the overall and progression-free survival of patients [2,3]. However, only a very small portion of the human kinome has been targeted with inhibitors at the phase I clinical trial level [4] and although many kinase inhibitors are currently in various phases of clinical trials for different cancers, there is a need for new inhibitors targeting novel kinases implicated in tumorigenesis, recurrence, and metastasis.

Doublecortin-like kinase 1 (DCLK1) is a microtubule-binding member of the calmodulin-dependent kinase family and has been identified as a tuft cell marker with stem-like properties in the small intestine and pancreas [5-10]. DCLK1 is overexpressed in tumors and pancreatic intraepithelial (PanIN) lesions of P48^Cre^Kras^LSLG12D^, Pdx1^Cre^; Kras^LSLG12D^, Pdx1^Cre^; Kras^LSLG12D^; Tp53^Flox/+^ and Mist1^CreER^; Kras^LSLG12D^ pancreatic cancer mice as well as surgical resection specimens of human pancreatic ductal adenocarcinoma (PDAC) patients, and is significantly correlated to PanIN lesion stage [8,9]. DCLK1 is also overexpressed in the Apc^min/+^ mouse model of intestinal neoplasia and surgical specimens of human colon cancer [5,7]. Recently, cutting-edge studies using the Dclk1^CreERT2^; Apc^min/+^ lineage tracing mouse model have demonstrated that Dclk1^+^ cells selectively mark tumor stem cells (TSCs) in intestinal adenomas and diphtheria-toxin inducible ablation of these cells results in massive loss of polyps with no apparent negative effects on the normal intestine [11]. Moreover, a recent study demonstrated that a unique population of DCLK1^+^ stem-like cells is capable of initiating pancreatic tumorigenesis [9]. These data provide a basis for DCLK1 targeted therapies.

DCLK1 has been targeted on the genetic level in some cancers with promising results. siRNA-mediated silencing of DCLK1 triggers apoptosis in SHSY5Y neuroblastoma cells [12]. Moreover, a recent study demonstrated that doxycycline-inducible knockdown of DCLK1 inhibits proliferation, mitochondrial activity, and ATP synthesis in N1E-115 neuroblastoma cells and delays progression of N1E-115 tumor xenografts [13]. Therapeutic targeting of DCLK1 in gastrointestinal cancer is highly desirable because of its expansion in tumors and tumor stem cell status. siRNA-mediated knockdown of DCLK1 in the AsPC-1 pancreatic cancer cell line results in inhibition of epithelial-to-mesenchymal transition (EMT) and oncogenic targets through induction of tumor suppressor miRNAs let-7a and miR-144 and EMT-inhibitor miR-200a [8]. In HCT116 (colon) and AsPC-1 (pancreatic) tumor xenografts, DCLK1 siRNA nanoparticle treatment significantly reduces tumor growth and inhibits pluripotency and angiogenic factors without any indication of toxicity [14,15]. Despite these compelling findings, the effect of inhibiting DCLK1 kinase activity has not been investigated in cancer. Recently, the Gray group developed a kinase inhibitor targeting Leucine-rich repeat kinase 2 (LRRK2), which is implicated in both genetically predisposed and sporadic Parkinson’s disease [16]. This compound, LRRK2-IN-1, displayed significant and relatively selective affinity for DCLK1 (K_d_ = 5 nM), compared to a K_d_ of 20 nM for LRRK2 [17]. Here we demonstrate that LRRK2-IN-1 elicits anticancer activity in part through inhibition of DCLK1, suggesting that DCLK1 kinase may be a promising anticancer target.

## Results

### LRRK2-IN-1 inhibits DCLK1 kinase activity

**Figure 1:**
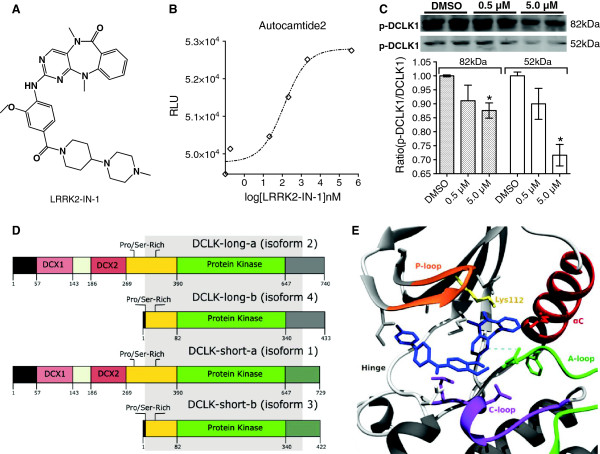
**LRRK2-IN-1 inhibits DCLK1 kinase activity.** An *in vitro* kinase assay was performed using Purified active DCLK1 kinase (0.25 μg) with 2.5 μg of autocamtide II substrate, 1 μM ATP, and either DMSO, 0.6, 2.5, 5, 10, or 50 nM LRRK2-IN-1 **(A)**. Using relative luminescent units (RLU) data, a sigmoidal-dose response curve was plotted in GraphPad Prism 6.0 (adj. R^2^ = 0.952) revealing an IC_50_ value of 2.61 nM **(B)**. AsPC-1 cells were treated with LRRK2-IN-1 at varying concentrations for 48 h. Following treatment cells were lysed, protein was isolated and quantified by BCA assay, and immunoblotting was performed with α-phospho-DCLK1. The ratio of phospho-DCLK1 to total DCLK1 (Figure 4B; 48 h) was determined and demonstrated decreased phosphorylation of DCLK1 (p < 0.05) following treatment **(C)**. Schematic demonstrating the shared protein kinase domain between DCLK1 isoforms referenced in Uniprot [Swiss-Prot: O15075] **(D)**. Three dimensional view of LRRK2-IN-1 binding site in DCLK-long-β revealing predicted interactions with residues of the hinge region, catalytic loop (*C*-*loop*), activation loop (*A*-*loop*), αC-helix (*αC*), and the highly conserved lysine (*Lys112*) of the kinase catalytic domain suggesting that LRRK2-IN-1 competes with ATP for the DCLK1 kinase binding pocket. Dashed lines mark the hydrogen bond formed with the conserved aspartate (“D” of the “DFG” motif) of the activation loop **(E)**.

Kinome profiling suggests that LRRK2-IN-1 (Figure 1A) inhibits DCLK1 kinase with a dissociation constant of 5 nM [17]. In order to confirm this inhibition we performed an *in vitro* kinase assay using commercially available purified DCLK1 protein and autocamtide2 substrate with low concentration ATP (1 μM). Remaining ATP following the reaction was quantified using luminescent kinase-glo® reagents which provides an inverse measure of kinase activity. Using this assay we estimated the IC_50_ of LRRK2-IN-1 inhibition of DCLK1 to be 2.61 nM (Figure 1B), supporting the previously reported kinome profiling results [17]. To assess the inhibition of DCLK1 phosphorylation *in vitro*, AsPC-1 cells were treated with LRRK2-in-1 for 48 h. Phospho-DCLK1 (Ser30/336) was decreased in both 52 and 82 kDa isoforms (long-β/α respectively) with LRRK2-IN-1 treatment in a dose-dependent manner. Quantification of the ratio of phospho-DCLK1/DCLK1 revealed that the 52 and 82 kDa isoforms decreased approximately 30% and 12.5% respectively following 5 μM LRRK2-in-1 treatment (p < 0.046; Figure 1C).

### LRRK2-IN-1 is an ATP-competitive inhibitor of DCLK1 kinase

Following confirmation of LRRK2-IN-1’s inhibitory activity against DCLK1 kinase, *in silico* molecular modeling and docking was conducted to determine the mechanism and localization of inhibition. Because the full crystal structure of DCLK1 has not been determined, homology models were constructed for DCLK1 isoform 2 (DCLK-long-α) and 4 (DCLK-long-β). The protein kinase domain is a highly conserved structural feature of all kinases and DCLK1 is a member of the calmodulin-dependent protein kinase (CAMK) family, which has many structures solved (Additional file 1: Figure S1A). Therefore, these models are expected to be reasonably accurate. Both Sparks^X^ Fold Recognition and SwissModel generated similar homology models of DCLK1 with a root mean square deviation (RMSD) of 0.89 Å, while the RMSD in the kinase domains of the long form models was 0.37 Å. Docking simulations were conducted using PatchDock and the homology model of DCLK-long-β, 81% of which encompasses the protein kinase domain shared by all DCLK1 isoforms (Figure 1D). In the kinase domain, the highest ranked docking site for LRRK2-IN-1 was located directly within the ATP-binding pocket with close proximity to the kinase hinge and interacting residues located in the catalytic loop, activation loop, glycine-rich loop (P-loop), and αC-helix and including the highly conserved, catalytic site lysine 112/419 (Figure 1E; Additional file 1: Figure S2A). These results suggest that LRRK2-IN-1 inhibits DCLK1 kinase activity by competing with ATP for the binding pocket.

### LRRK2-IN-1 inhibits proliferation, migration, and induces cell death with hallmarks of apoptosis

**Figure 2:**
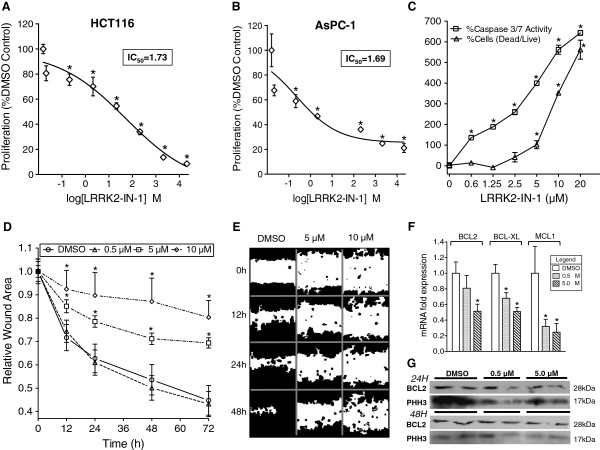
**LRRK2-IN-1 elicits anticancer activity *in vitro*.** HCT116 and AsPC-1 cells were seeded into 96-well plates at 10^4^ cells per well and allowed to attach overnight at 37°C. LRRK2-IN-1 was added in triplicate to the wells at concentrations of 0 (DMSO), 0.3, 0.6, 1.25, 2.5, 5, 10, and 20 μM and incubated at 37°C. After 48 h an MTT proliferation assay was performed and revealed significant inhibition of cell proliferation starting at 0.62 μM **(A-B)**. A live/dead assay in AsPC-1 cells was performed 24 h post treatment to confirm LRRK2-IN-1’s cytotoxic effect. Additionally, a luminescent assay revealed that this effect co-ocurred with Caspase 3/7 activation, both p < 0.0001 by ANOVA **(C)**. AsPC-1 cells were seeded into 6-well plates allowed to reach confluence, scratched, and treated with DMSO, 0.5, 5, or 10 μM LRRK2-IN-1 and incubated at 37°C. The wound area was imaged at baseline, 12, 24, 48, and 72 h and quantified using ImageJ **(D-E)**. mRNA expression analysis of AsPC-1 cells treated with DMSO or LRRK2-IN-1 at concentrations of 0.5 μM or 5.0 μM for 8 h revealed a significant downregulation (p < 0.01) of apoptosis regulators BCL2, BCL2L1 (BCL-XL), and MCL1 mRNA expression **(F)**. Western blots showed decreased BCL2 and Phosphohistone H3 at both 24 and 48 h post LRRK2-IN-1 treatment **(G)**.

DCLK1 is overexpressed or demonstrates strong expression in many colon and pancreatic cancer cell lines (Additional file 1: Figure S2C) [18,19]. To assess the functional effects of LRRK2-IN-1 *in vitro* we chose to focus on the AsPC-1 human pancreatic cancer and HCT116 human colon cancer cell lines, which are both well characterized for their DCLK1 expression in the literature [7,9,14,15,20-22]. Both AsPC-1 and HCT116 cells were treated with various concentrations of LRRK2-IN-1 for 48 h and MTT proliferation assays were conducted. A significant dose-dependent reduction of cell proliferation was observed in the highly proliferative HCT116 colon cancer cell line (Figure 2A) and the AsPC-1 pancreatic cancer cell line (Figure 2B). Fitting a sigmoidal-dose response curve revealed IC_50_ values of 1.69 and 1.73 μM for AsPC-1 (R^2^ = 0.79) and HCT116 (R^2^ = 0.94) cell lines respectively. Moreover, this anti-proliferative activity was observed in DLD-1 and HT-29 colon cancer cells and MiaPaCa-2 and SW1990 pancreatic cancer cells. Notably, SW1990 cells, which express high levels of DCLK1 (Additional file 1: Figure S2C), displayed resistance to LRRK2-IN-1 compared to the other lines with an IC_50_ of >5 μM (Additional file 1: Figure S1B). Furthermore, LRRK2-IN-1 was found to have cytotoxic effects in the AsPC-1 cell line by live/dead viability assay 24 h post treatment (Figure 2C; Additional file 1: Figure S2B), and cells at this time point demonstrated significant dose-dependent increases in caspase-3/7 activity (Figure 2C), which was exponentially associated (R^2^ = 0.98) with loss of cell viability (Additional file 1: Figure S2D). Moreover, LRRK2-IN-1 inhibited AsPC-1 migration at 5 and 10 μM starting after 12 h treatment (Figure 2D-E). Relative to the original wound area, the average wound area was approximately 45% 72 h post DMSO treatment, but was approximately 70% and 80% at this time point for 5 and 10 μM LRRK2-in-1 treatment respectively. This anti-migratory effect was highly dose dependent (p < 0.0001 by ANOVA). Additionally, gene expression levels of apoptosis pathway inhibitors, BCL2L1 (BCL-XL), BCL2, and MCL1, were significantly decreased in AsPC-1 cells treated with LRRK2-IN-1 for 8 h (Figure 2F). In confirmation of LRRK2-IN-1’s anti-proliferative and pro-apoptotic properties, mitotic marker phosphohistone H3 and anti-apoptotic marker BCL2 protein levels were inhibited 24 and 48 h post treatment in these cells (Figure 2G).

### LRRK2-IN-1 induces G1 and G2/M cell cycle arrest

**Figure 3:**
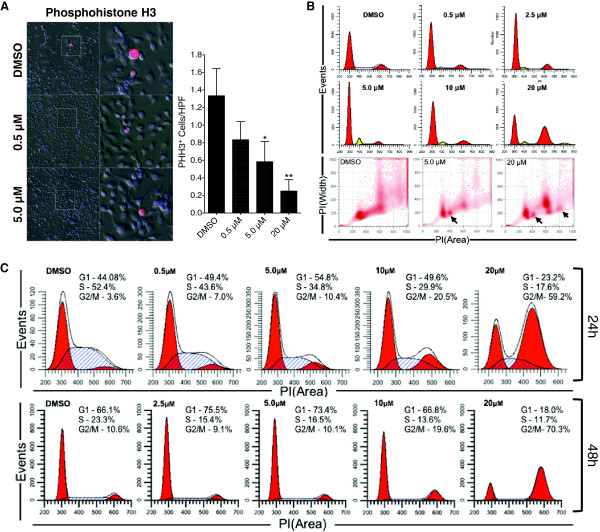
**LRRK2-IN-1 induces G1 and G2 arrest *in vitro*.** Immunofluorescence of AsPC-1 cells treated with LRRK2-IN-1 demonstrating a decrease in mitotic cells at 48 h as determined by phosphohistone H3 (PHH3) positive cells **(A)**. Cell cycle analysis of AsPC-1 cells 48 h post LRRK2-IN-1 demonstrating maximum G1 arrest at 5.0 μM and G2/M arrest at 20 μM regardless of ploidy level. Yellow peaks denote aneuploid populations and arrows identify these populations in the density maps **(B)**. Cell cycle analysis of AsPC-1 cells 24 and 48 h post LRRK2-IN-1 treatment demonstrating G1 arrest at low doses and potent G2/M arrest at higher doses **(C)**.

AsPC-1 cells were treated with LRRK2-IN-1 for 24 and 48 h. Cell cycle analyses were performed and revealed that LRRK2-IN-1 induces G1 arrest at low doses (5 μM) and G2/M arrest at high doses (20 μM) at both time points (Figure 3C). Moreover, G2/M arrest was strongly correlated to loss of cell viability at 24 h (Additional file 1: Figure S2E). Aneuploidy is well known to contribute to drug resistance [23]. LRRK2-IN-1 also induced G1 and G2/M arrest in aneuploid AsPC-1 cells as demonstrated by PI analysis (Figure 3B). The dose-dependent decrease in phosphohistone H3 expression (p = 0.013 by ANOVA) following treatment suggests arrest of cells in G2 phase specifically at higher doses (Figures 3A & 2G). These data demonstrate that LRRK2-IN-1 reduces cancer cell proliferation and viability by inducing both G1 and G2/M arrest. Moreover, the divergent cell cycle status following low and high-dose LRRK2-IN-1 suggests that as drug concentration increases further inhibition of target kinase activity, inhibition of a greater number of kinases, or another downstream interaction causes cells to preferentially pool in G2/M phase.

### LRRK2-IN-1 inhibits DCLK1 mRNA and protein expression

**Figure 4:**
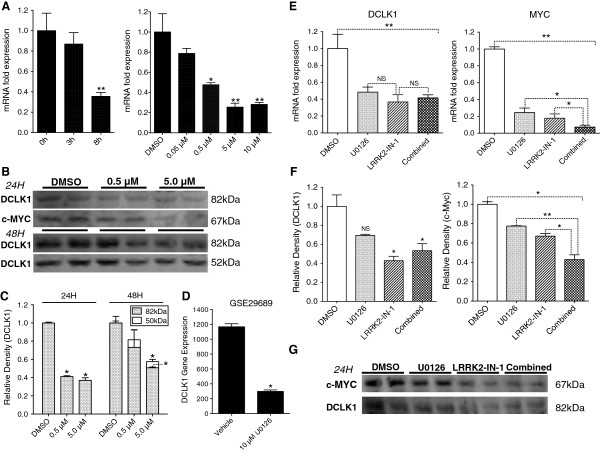
**LRRK2-IN-1 downregulates DCLK1 expression and downstream target c-Myc.** LRRK2-in-1 downregulates DCLK1 mRNA expression in a dose and time-dependent manner in AsPC-1 cells **(A)**. LRRK2-IN-1 significantly downregulates DCLK1 protein expression at 24 and 48 h and downregulates protein expression of DCLK1 downstream target c-MYC **(B-C)**. NCBI GEO data demonstrating inhibition of MAPK7/ERK5 with 10 μM U0126 resulting in downregulation of DCLK1 gene expression in SW480 cells **(D)**. Treatment of AsPC-1 cells with 10 μM U0126 downregulates DCLK1 as well as shared downstream target c-MYC’s mRNA and protein levels. However, whereas combined treatment with this dose of U0126 (10 min pretreatment) and 5 μM LRRK2-IN-1 results in synergistic downregulation of c-MYC, DCLK1 is unaffected or slightly upregulated suggesting that U0126 and LRRK2-IN-1 may regulate DCLK1 mRNA and protein expression by the same mechanism of action **(E-G)**.

Following LRRK2-IN-1 treatment, DCLK1 mRNA levels were decreased in a time-dependent manner when treated with 5 μM of LRRK2-IN-1 (p = 0.004 by ANOVA), and in a dose-dependent manner when treated with various concentrations of LRRK2-IN-1 for 8 h (p < 0.0001 by ANOVA; Figure 4A). DCLK1 mRNA levels were consistently decreased approximately 65-75% after 5 μM LRRK2-IN-1 treatment for 8 h. DCLK1 protein levels were also decreased in a dose-dependent manner, with an approximately 60% reduction for the 82 kDa isoform at 24 h, and a 45% reduction at 48 h following 5 μM LRRK2-IN-1 treatment (p < 0.03; Figure 4B-C). Downstream target c-MYC protein expression was also downregulated 24 h following treatment (Figure 4B). We hypothesized that DCLK1 mRNA and protein expression is regulated through the inhibition of another target of LRRK2-IN-1. The MAPK/ERK signaling pathway is an important regulator of many cellular functions and modulates a wide range of molecular targets. MAPK7 (ERK5) is inhibited by LRRK2-IN-1 (K_d_ = 28 nM) [17], so we investigated whether this may be a feasible driver of the observed downregulation of DCLK1 gene and protein expression. U0126 was developed as an inhibitor of MEK1/2 but has also been shown to inhibit MAPK7 [24]. Moreover, it was previously reported that U0126 suppresses upregulation of DCLK1 in response to NGF stimulation in PC12 cells [25] and a search of the NCBI gene expression omnibus database produced multiple datasets demonstrating significant DCLK1 downregulation following U0126 inhibition in colon cancer cell lines (Figure 4D & Additional file 1: Figure S2F) [26,27]. As predicted, DCLK1 mRNA levels were decreased after 10 μM U0126 treatment (Figure 4E). Ten minutes pretreatment of cells with this dose of U0126 followed by 5 μM LRRK2-IN-1 treatment caused no further decrease in DCLK1 gene expression (Figure 4E). However, MYC, which is downstream of both DCLK1 [8] and MAPK7 and encodes the c-MYC protein, was synergistically downregulated with combinatorial drug treatment compared to LRRK2-IN-1 or U0126 treatment alone (p < 0.005; Figure 4E). Highly similar results were also observed for protein expression of both DCLK1 and c-MYC 24 h post treatment (p < 0.05; Figure 4F-G). These results suggest that MAPK7 inhibition is a likely mechanism of action for DCLK1 downregulation by LRRK2-IN-1. Moreover, this data suggests that DCLK1 and the MEK1/2-MAPK7 cascade regulate c-MYC expression by complementary mechanisms.

### LRRK2-IN-1 reduces pluripotency and stem cell associated gene expression

**Figure 5:**
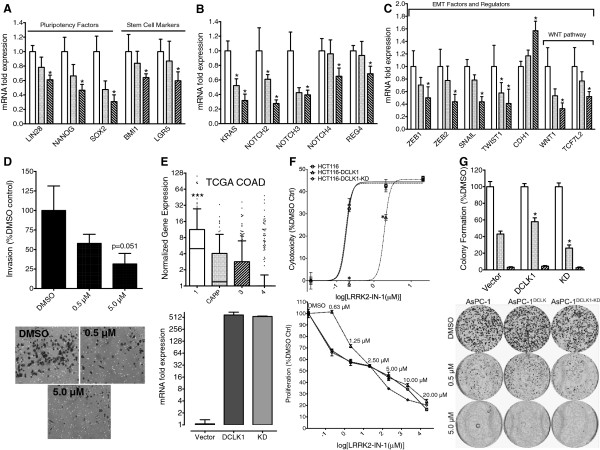
**LRRK2-IN-1 induces anti-oncogenic molecular changes and LRRK2-IN-1 induced cell death depends on DCLK1 kinase activity.** AsPC-1 cells treated with LRRK2-IN-1 show decreased expression of stem **(A)**, oncogenic **(B)**, and EMT-related gene expression **(C)** (Vehicle = white; 0.5 μM = gray; 5.0 μM = striped bars. p < 0.05). Consistent with the changes seen in EMT-related gene expression, LRRK2-IN-1 significantly decreases invasion in AsPC-1 cells at 5 μM **(D)**. DCLK1 isoform 1 is the most highly expressed referenced variant in colon cancer (**E** – *top panel*). Lentiviral overexpression of this isoform and its kinase dead form demonstrates approximately equal levels of DCLK1 transcript as determined by real-time RT-PCR of infected HCT116 cells (**E** – *bottom panel*). HCT116(vector), HCT116-DCLK1, and HCT116-DCLK1-KD cell lines were treated with LRRK2-IN-1 at various concentrations. After 48 h an MTT proliferation assay was performed. Compared to vector control cells, HCT116-DCLK1-KD cells demonstrated no change from 0.63 – 2.5 μM and decreased resistance at 5 and 10 μM, while HCT116-DCLK1 cells demonstrated significantly increased resistance to LRRK2-IN-1 cytotoxicity at 0.63 and 1.25 μM concentrations leading to a shift in relative cytotoxicity at low doses **(F)**. AsPC-1(vector), AsPC-1-DCLK1, and AsPC-1-DCLK1-KD cells were treated with vehicle, 0.5, or 5 μM of LRRK2-IN-1 and allowed to form colonies for 9 days. Colonies were stained with crystal violet and plates were divided into grids and counted. AsPC-1-DCLK1 cells demonstrated significant resistance at 0.5 μM compared to vector control cells, while AsPC-1-DCLK1-KD cells demonstrated a significant decrease in resistance compared to vector control cells **(G)**.

Stem and stem-like cells are characterized by the expression of specific transcription factors termed pluripotency factors. We have recently demonstrated that knockdown of DCLK1 decreases expression of these factors through regulation of miR-145 [15]. Expression levels of LIN28, NANOG, and SOX2 were significantly reduced after LRRK2-IN-1 treatment in AsPC-1 cells (Figure 5A). LGR5 and BMI1 are stem-cell markers coexpressed with DCLK1 in the intestine [28,29], regulated downstream of DCLK1 following radiation injury in intestinal epithelial DCLK1 knockout mice [30], and markers of populations of cells with tumor stem cell activity in intestinal adenomas [31] and pancreatic adenocarcinoma [32]. Both LGR5 and BMI1 mRNA expression levels were significantly downregulated following LRRK2-IN-1 treatment (Figure 5A). These results along with the c-MYC expression results demonstrate that LRRK2-IN-1 possesses anti-stemness properties.

### LRRK2-IN-1 inhibits oncogenic and EMT marker gene-expression

Mutation of the KRAS oncogene is the primary initiator of PDAC and exerts its influence through multiple pro-tumorigenic pathways [33]. These mutations are also present with high frequency in colorectal cancer and are correlated with poor overall survival [34]. LRRK2-IN-1 caused a significant downregulation of KRAS expression in AsPC-1 cells. Additionally, significant decreases were found in PDAC related genes NOTCH2-4 and REG4 (Figure 5B). However, there was no effect on NOTCH1 expression (data not shown).

At the time of diagnosis most PDAC patients present with metastatic disease [35]. Metastasis is driven by the process of EMT under the control of specific transcription factors, which allow newly transformed cancer cells to gain a stem-cell like phenotype [36]. LRRK2-IN-1 significantly upregulated epithelial marker E-cadherin (CDH1) while downregulating EMT factors SNAI1 (SNAIL), ZEB1, ZEB2, and TWIST1 mRNA expression (Figures 4C). The WNT pathway is heavily involved in regulating EMT primarily through nuclear β-catenin [37]. LRRK2-IN-1 downregulated the expression of WNT pathway genes WNT1, one of the upstream signaling proteins of the pathway, and TCF7L2/TCF4 (Figure 5C), an essential transcription factor which in complex with β-catenin directly regulates ZEB1 expression [38]. Consistent with the molecular changes seen in EMT related gene and protein expression there was a dose-dependent decrease in the invasive potential of AsPC-1 cells 24 h post LRRK2-IN-1 treatment, with an approximately 70% reduction in invasive potential following 5 μM treatment (Figure 5D).

### DCLK1 promotes resistance to LRRK2-IN-1

Analysis of RNA deep-sequencing data from the cancer genome atlas colon cancer dataset [39] revealed that DCLK1 transcript variant 1, which encodes isoform 1 (also known as DCLK-short-α), is the most highly expressed annotated variant (Figure 5E). To demonstrate that DCLK1 is a significant factor in LRRK2-IN-1’s anticancer properties, both HCT116 and AsPC-1 cells were infected with Lentivirus containing the cDNA of DCLK1 isoform 1 or a kinase-dead mutant (K419R) of this isoform, which was previously shown to inactivate DCLK1’s kinase activity [40-42]. DCLK1 mRNA expression levels were increased about 500 fold in both wild type DCLK1 and DCLK1-K419R overexpressing cells compared to vector infected cells (Figure 5E). DCLK1 has recently been reported to be a significant contributor to proliferation and mitochondrial activity in neuroblastoma cells [13]. We found that overexpression of DCLK1 resulted in significant resistance to LRRK2-IN-1’s anti-proliferative effect in the highly proliferative HCT116 cell line up to 2.5 μM (p = 0.03), whereas overexpression of DCLK1-K419R was virtually identical to vector control at these doses (Figure 5F). To further characterize DCLK1-based resistance to LRRK2-IN-1 we performed a colony formation assay using AsPC-1 cells overexpressing DCLK1 and DCLK1-K419R. We observed a significant increase in colonies formed relative to vector control cells in cells overexpressing DCLK1 at 0.5 μM (p = 0.035), while cells overexpressing DCLK1-K419R had fewer colonies at this concentration. 5 μM LRRK2-IN-1 proved to be highly effective and colonies were virtually non-existent in all cell lines (Figure 5G). These results demonstrate that DCLK1 kinase activity confers resistance to LRRK2-IN-1.

### LRRK2-IN-1 inhibits the growth of pancreatic tumor xenografts

**Figure 6:**
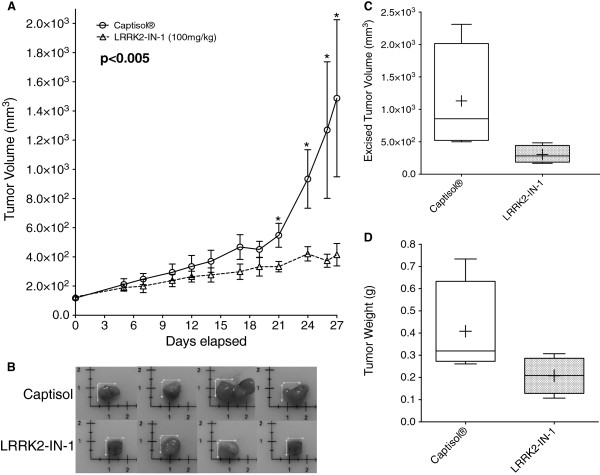
**LRRK2-IN-1 is effective *in *vivo.** AsPC-1 cells (0.5×10^6^) were injected subcutaneously into the flanks of athymic nude mice (n = 4) and allowed to grow until the tumor reached an average volume of 100 mm^3^. LRRK2-IN-1 was solubilized in 20% Captisol® and xenografts were injected peritumorally with either 20% Captisol (vehicle) or LRRK2-IN-1 (100 mg/kg) for a total of 12 injections. LRRK2-IN-1 treatment resulted in a significant decrease in tumor volume as a function of time **(A)** and reduced final excised tumor volume (p = 0.09; **B-C**) and tumor weight (p = 0.14; **D**). The + symbol denotes the mean.

To assess the effect of LRRK2-IN-1 *in vivo*, a tumor xenograft study (n = 4 per group) was conducted. AsPC-1 tumor xenografts were injected with LRRK2-IN-1. Peritumoral injection of the drug resulted in a significant decrease in tumor volume (Figure 6A). There was also a notable decrease in the excised volume and weight of tumors from LRRK2-IN-1 treated mice (Figure 6B-D). Both the mean excised tumor volume and weight decreased more than 50% following LRRK2-IN-1 treatment for 4 weeks. This data demonstrates that LRRK2-IN-1 is effective *in vivo*.

## Discussion

The functional significance of DCLK1 in cancer has only been explored recently. Although siRNA-mediated knockdown of DCLK1 gene expression is antiproliferative and induces tumor growth arrest [7,11,13-15], the effect of inhibiting DCLK1 kinase activity in cancer had not been previously assessed. The findings presented here have potentially wide-ranging implications in the development of novel, targeted agents against DCLK1 and provide additional validation for DCLK1 as a therapeutic target. It is notable that some novel anti-cancer kinase inhibitors such as MAPK7 inhibitor XMD8-92, PLK1-inhibitor BI-2536, and ALK-inhibitor TAE-684 [43] demonstrate affinity for DCLK1 kinase that is comparable to their target kinases (http://lincs.hms.harvard.edu/). This off-target inhibition may play a role in the therapeutic effects of these drugs. In fact, DCLK1 gene expression was highly upregulated in 2 out of 4 HCT116 cell clones that demonstrated resistance to BI-2536 [44]. The structural similarities between these inhibitors and others that have been synthesized and profiled may aid in the design of DCLK1 inhibitors with improved chemical characteristics and favorable delivery and safety profiles. Additionally, the results of our study suggest the possibility that DCLK1 kinase activity may be involved in regulating proliferation, cell cycle, EMT, and stemness pathways in cancer. However, before these hypotheses can be confirmed, it will be essential to carefully dissect the role of each domain in each of the 4 primary human DCLK1 isoforms in addition to the smaller peptide-like isoform of DCLK1 (CAMK-related peptide; “CARP”).

The MAPK/ERK pathway regulates many functions essential to cancer cell survival and proliferation [45]. In these studies we used U0126 to demonstrate a link between MEK1/2 cascade inhibition and DCLK1 expression in human pancreatic cancer cells and confirmed our results with microarray data generated by other groups in treated colon cancer cell lines (Figure 4D & Additional file 1: Figure S2F). Additionally, a previous paper demonstrated that nuclear translocation of phosphorylated DCLK1-short is attenuated in U0126-treated *Xaenopus laevis* melanotrope cells [46], which if broadly applicable, implies that MEK1/2 cascade inhibition has the potential to limit or prevent DCLK1-based intracellular signaling. The data from this study and our previous studies [7,8] suggest that DCLK1 interacts with and regulates the MAPK/ERK effector c-MYC. Interestingly, a large-scale protein-protein interaction study identified physical interaction between DCLK1 and c-MYC at DCLK1 amino acids 385–399 [47], located at the N-terminal end of DCLK1’s kinase domain. If confirmed, this would suggest that targeting DCLK1 regulates MYC through post-translational modification, which could also explain the synergistic effect of combinatorial LRRK2-IN-1 and U0126 treatment on this target.

## Conclusions

Although the overall function of DCLK1 and it’s multiple isoforms and functional domains remains to be elucidated, our studies and others have implicated this protein as a key regulator of gut injury response and as a major regulator of proliferative, angiogenic, stemness, and EMT pathways in cancer. Taken together these findings support DCLK1’s role as a broad regulator of the molecular and cellular mechanisms of tumorigenesis. Moreover, the restricted expression of DCLK1 in a limited set of normal cells and its upregulation and tumor-stem cell status in cancer tissue make it an ideal target for cancer therapy. The specificity of LRRK2-IN-1 for DCLK1 and the results of our studies support the development of DCLK1 kinase inhibitors against cancer. Given the recalcitrance of advanced gastrointestinal cancers in general, this therapeutic concept may have the potential to overcome limitations of current therapies which only target proliferating cells in the primary tumor, by repressing the EMT processes and stemness characteristics that fuel the fatal metastatic and drug resistant characteristics of advanced tumors.

## Methods

### *In vitro* kinase assay

Purified kinase-active DCLK1 (0.25 μg, Signalchem) was incubated in kinase buffer II (Alfa Aesar) with 2.5 μg of autocamtide II substrate (American Peptide Co.), 1 μM ATP (Sigma), and either DMSO, 0.6, 1.25, 2.5, 5, 10, or 50 nM LRRK2-IN-1 (Calbiochem) for 15 min at 30°C. Subsequently, Kinase-Glo® reagent (Promega) was added 1:1 to the reactions which were gently mixed and then incubated for another 15 minutes protected from light. Results were obtained using a Synergy HT plate reader (Biotek) capable of luminometric measurements.

### Molecular docking analysis

The ligand structure for LRRK2-IN-1 was constructed in ChemDraw (CambridgeSoft®) and energy-minimized with UCSF Chimera [48]. Homology models of DCLK1 isoform 2 (DCLK-long-α) and 4 (DCLK-long-β) were generated using Sparks^X^ Fold Recognition software [49]. Docking analyses were carried out with PatchDock [50] and results were visualized with UCSF Chimera and LigPlot + [51]. Model predictions were checked for reasonable precision by repeating the modeling procedures using SwissModel [52].

### Cell culture

AsPC-1, MiaPaCa-2, and SW1990 human pancreatic cancer cells, and HCT116, HT-29, and DLD-1 human colorectal cancer cells, were obtained from ATCC and grown in Dulbecco’s Modified Eagle’s Medium with 4.5 g/L glucose and L-glutamine, without sodium pyruvate (Cellgro) supplemented with 10% fetal bovine serum (Sigma) at 37°C and 5% CO_2_.

### Site-directed mutagenesis and lentiviral plasmid construction

Human DCLK1 isoform short-α cDNA tagged with turboGFP (tGFP) (Origene) was used to create a kinase-dead mutant, in which the lysine at residue 419 was substituted with an arginine by site-directed mutagenesis using the QuickChange II mutagenesis kit (Agilent Technologies). The mutant construct was confirmed by DNA sequencing. Both wild-type DCLK1-tGFP and K419R-tGFP cDNAs were then amplified and ligated into pCR8-GW-TopoD (Invitrogen) following the manufacturer’s protocol. Wild-type DCLK1-tGFP and K419R-tGFP cDNAs were then transferred to pLenti CMV PURO DEST empty (gift from Dr. Eric Campeau) using Clonase 2 (Invitrogen) following the manufacturer’s recommendations.

### Generation of lentiviral particles and cell lines

The expression plasmids constructed above were co-transfected along with packaging plasmids pMD2.G (Addgene), pMDL/RRE g/p (Addgene) and pRSV-Rev (Addgene) into 293 T cells. DNA was transfected into cells using the PEI transfection method with a PEI to DNA ratio of 1:1. Supernatants were harvested 48 and 72 h post-transfection and cleared through a 0.45 μm filter. These viral supernatants were concentrated using polyethylene glycol 8000 (Sigma-Aldrich) as previously described [53] and cells were infected with concentrated virus and selected with puromycin (Sigma-Aldrich) to establish stable cell lines.

### Cell proliferation assays

Cells (10^4^ cells per well) were seeded into a 96-well tissue culture plate in triplicate. The cells were cultured in the presence of LRRK2-IN-1 with DMSO as a vehicle at 0, 0.31, 0.63, 1, 2, and 5, 10, and 20 μM. 48 h post treatment, 10 μl of TACS MTT Reagent (RND Systems) was added to each well and the cells were incubated at 37°C until dark crystalline precipitate became visible in the cells. 100 μl of 266 mM NH_4_OH in DMSO [54] was then added to the wells and placed on a plate shaker at low speed for 1 minute. After shaking, the plate was allowed to incubate for 10 minutes protected from light and the OD_550_ for each well was read using a microplate reader. The results were averaged and calculated as a percentage of the DMSO (vehicle) control +/- the standard error of the mean.

### Live-dead and Caspase 3/7 activity assays

AsPC-1 cells were seeded into a 96-well plate at 10^4^ cells per well and allowed to attach overnight at 37°C. LRRK2-IN-1 was added to the wells at concentrations of 0 (DMSO), 0.3, 0.6, 1.25, 2.5, 5, 10, and 20 μM in duplicate and incubated at 37°C for 24 h. Fluorescent Live/Dead viability (Invitrogen) and luminescent CaspaseGlo® 3/7 activity (Promega) assays were performed according to the manufacturer’s protocol.

### Flow cytometry

To assess cell cycle status, AsPC-1 cells were treated with LRRK2-IN-1 for 24 or 48 h, trypsinized, centrifuged at 4°C, washed with Automacs rinsing solution (Miltenyi), and then fixed in 70% ethanol on ice for 30 minutes. Following fixation the cells were washed with Automacs rinsing solution and incubated with propidium iodide (50 μg/ml) and treated with RNAse A. In another experiment to analyze the cycling status of smaller subpopulations of cells this process was repeated with longer fixation (>2 h) and the addition of 0.1% Triton-X 100 for permeabilization. Data was collected on FACS Calibur and analyzed in ModFit LT.

### Matrigel transwell invasion assays

Control or matrigel coated transwells (BD Biosciences) were prepared by soaking in serum-free media for 2 h at 37°C in a 24-well plate. Subsequently, AsPC-1 cells (5000/well) were seeded into each transwell in serum-free media and treated with LRRK2-IN-1 or DMSO (vehicle) in duplicate. Cell culture medium containing 10% FBS was added to the bottom of each well as chemoattractant and the cells were incubated for 22 h. Afterwards, a cotton swab was used to scrape non-invasive/migratory cells off the top of transwells and the remaining cells were fixed with 100% methanol, stained with 1% toluidine blue/1% borax, and allowed to dry. After drying, the film from the transwell inserts was removed with a scalpel blade and mounted on slides. For each sample 5 fields were counted at 10X magnification. Percent invasion was calculated by dividing the number of invading cells (matrigel-coated inserts) by the number of migrating cells (control inserts) and multiplying by 100.

### Colony formation assay

AsPC-1 cells (5000/well) overexpressing DCLK1, DCLK1-K419R, or vector were seeded into 6 cm dishes in cell culture media containing 10% FBS and either DMSO or LRRK2-IN-1 at 0.5 or 5 μM. The cells were allowed to grow for 9 days and then washed with PBS, fixed with glacial acetic acid/methanol solution (1:3), and washed with PBS again. Colonies were stained with 0.5% crystal violet for 15 minutes and washed with tap water to remove excess stain. Colonies were then counted under a stereomicroscope using a 1 cm^2^ grid. Four squares from four quadrants were counted for each plate. The results were normalized to DMSO for each cell line in order to obtain the percent of colony formation. Stained colonies were then imaged, thresholded in ImageJ, and highlighted for visualization using the *find edges* ImageJ function.

### Wound healing assay

10^5^ AsPC-1 cells were seeded into 6-well plates and allowed to grow to confluence. A sterile 200 μl pipette tip was used to scratch the confluent layer, and detached cells were removed with washing. For each plate 9 points were selected and marked and baseline images were taken at 4× magnification. Following baseline imaging drug or vehicle was added to each well. Images were again taken at 12, 24, 48, and 72 h. The migrating edges were detected using the *find edges* function in ImageJ and images were converted to 16-bit format and thresholded. The empty region between migrating monolayer edges was selected and the area was measured using the *measure* tool. These values were used to perform standard calculations and migration over time was assessed by ANOVA.

### Quantitative real-time RT-PCR

AsPC-1 cells (10^5^/well) were seeded into 6-well plates in triplicate in the presence of LRRK2-IN-1 and incubated at 37**°**C for 8 h. The cells were lysed, and total RNA was isolated using Tri Reagent (MRC) per the manufacturer’s instructions. First strand cDNA synthesis was carried out using SuperScript II Reverse Transcriptase and random hexanucleotide primers (Invitrogen). The complementary DNA was subsequently used to perform RT-PCR on an iCycler IQ5 Thermal Cycler (BioRad) using SYBR Green (Molecular Probes) with gene-specific primers and JumpStart™ Taq DNA polymerase (Sigma). The crossing threshold value assessed was normalized to β-actin and quantitative changes in mRNA were expressed as fold-change relative to control ± SEM value. The Student’s t-test was used to determine statistical significance. The primer sequences for the genes analyzed are provided in Additional file 1: Table S1.

### Generation of phospho-DCLK1 antibody

A rabbit polyclonal antibody was commercially generated (Abbomax) to target ser-30(β)/336(α), which has previously been demonstrated to be phosphorylated, against a 15 amino acid peptide sequence similar to others previously used to generate phospho-DCLK1 antibodies [46,55]. To confirm specificity, ELISA was performed using phosphospecific and non-phosphospecific peptide.

### Western blotting

Denatured proteins of cell lysates were subjected to Western blot analysis. The concentration of total proteins was determined by BCA protein assay (Pierce, Rockford, IL). 40 μg of total proteins was separated on a 7.5%–15% SDS polyacrylamide gel and transferred onto a PVDF membrane. The membrane was blocked in 5% non-fat dry milk for 1 h and probed overnight with primary antibody. Subsequently the membrane was incubated with infrared cw800-conjugated secondary antibody for 1 h at room temperature. The proteins were detected using a LICOR Odyssey Infrared Imager. Protein density quantification was performed in Image Studio Lite (LICOR). The antibodies used were α-DCLK1 (ABCAM, AB31704), α-phosphoDCLK1, α-C-MYC (Santa Cruz, SC-40), α-BCL2 (Santa Cruz, SC-492), and α-phosphohistone H3 (EMD-Millipore 07–492).

### Immunofluorescence

Cells grown on glass coverslips and treated with DMSO or LRRK2-IN-1 were rinsed with PBS, fixed in formalin, rinsed again with PBS, and permeabilized with 0.1% Triton-X. Following permeabilization cells were incubated at 4°C overnight with α-phosphohistone H3 diluted 1:10000 in 0.01% Triton-X containing 1% BSA. After incubation, cells were washed with PBS, and secondary antibody (Alexa-Fluor 547) diluted 1:2000 in PBS was added and allowed to incubate for 30 minutes at room temperature. After washing with PBS the coverslips were counterstained with Hoechst 33342, rinsed with PBS and distilled water, mounted on slides using Prolong gold antifade reagent (Invitrogen), and imaged with a Nikon Eclipse Ti microscope.

### Xenograft tumor study

AsPC-1 cells (5×10^5^) were injected subcutaneously into the flanks of athymic nude mice and allowed to grow until the tumor reached an average volume of 100 mm^3^. LRRK2-IN-1 was solubilized in 20% Captisol® and the xenografts were injected peritumorally with either 20% Captisol (vehicle) or LRRK2-IN-1 (100 mg/kg). Injections were performed on Monday, Wednesday, and Friday for 4 weeks (a total of 12 injections). Horizontal and vertical tumor diameter was measured on each injection date with calipers and tumor volume was calculated using the formula: *tumor volume* = *0.5 × length × width*^*2*^. At the end of the injection period mice were killed by CO_2_ asphyxiation and tumors were excised, weighed, and measured. All animal experiments were performed in accordance with standards set forth by the University of Oklahoma Health Sciences Center’s Institutional Animal Care and Use Committee.

### Analysis of TCGA and NCBI GEO datasets

To perform The Cancer Genome Atlas (TCGA) Colon Adenocarcinoma (COAD) dataset analysis, isoform specific RNA-seq results for all stages of colon adenocarcinoma were downloaded from the TCGA open-access server. A workflow in Knime (version 2.8.1) was used to identify and aggregate transcript data. NCBI gene expression omnibus (GEO) datasets were downloaded using NCBI’s built in GEO2R analysis tool.

### Statistical analysis

All statistical analyses were performed using Graphpad Prism 6.0 and Microsoft Excel. One-way ANOVA and the Student’s T-test were used to determine statistical significance unless otherwise noted. For all analyses p < 0.05 was considered to be statistically significant.

## Competing interests

C.W. Houchen is a Co-Founder of COARE Biotechnology. The other authors disclose no potential conflicts of interest.

## Authors’ contributions

NW, DQ, RM, and CWH conceived of the study, designed the experiments, interpreted the data, and drafted the manuscript. NW, DQ, RM, and DBO performed the *in vitro*, *in vivo*, and histological procedures, collected the data, and prepared the images for publication. WLB and RJ aided in experimental design, prepared lentivirus, generated stable cell lines, provided technical support, and proofread the manuscript. PC, SMS, and NA provided technical support and proofread the manuscript. All authors read and approved the final manuscript.

## Supplementary Material

Additional file 1: Table S1 Primers sequence for genes analyzed by real-time RT-PCR. **Figure S1.** Cladogram of proteins closely related to DCLK1 as determined by ClustalΩ using the human kinome as input. Highlighting denotes solved structures **(A)** Anti- proliferative effect of LRRK2-IN-1 on colon and pancreatic cancer cell lines 48 h post-treatment **(B)**. **Figure S2.** Two-dimensional plot of LRRK2-IN-1 and interacting residues as plotted by LigPlot and color-coded to agree with Figure 1E Red rays denote hydrophobic interactions and the green dashed line denotes 2.63A length hydrogen bonding between the ligand and Aspartate 226. Residue labeling agrees with the SwissProt entry for DCLK1 isoform 4 **(A)**. Fluorescent images of cells stained with Calcein-AM (Live/Green) and Eth-D (Dead/Red) following LRRK2-IN-1 treatment **(B)**. NCBI Geo data of DCLK1 gene expression in various pancreatic cancer cell lines compared to HPDE immortalized normal human pancreatic ductal epithelial cells (GSE40099) and comparison of DCLK1 gene expression in colon cancer cell lines from the NCI-60 cell panel (GDS 1761) **(C)** Exponential plots demonstrating strong associations between cell death and caspase activity and percentage of G2/M arrest **(D-E)** NCBI Geo data of DCLK1 gene expression in colon cancer cell lines treated with 10 μM of U0126 **(F)**. Click here for file

## References

[B1] Gambacorti-Passerini C Antolini L Mahon FX Guilhot F Deininger M Fava C Nagler A Della Casa CM Morra E Abruzzese E D'Emilio A Stagno F le Coutre P Hurtado-Monroy R Santini V Martino B Pane F Piccin A Giraldo P Assouline S Durosinmi MA Leeksma O Pogliani EM Puttini M Jang E Reiffers J Valsecchi MG Kim DW Multicenter independent assessment of outcomes in chronic myeloid leukemia patients treated with imatinib J Natl Cancer Inst 2011 103 553 561 10.1093/jnci/djr060 21422402

[B2] Llovet JM Ricci S Mazzaferro V Hilgard P Gane E Blanc JF de Oliveira AC Santoro A Raoul JL Forner A Schwartz M Porta C Zeuzem S Bolondi L Greten TF Galle PR Seitz JF Borbath I Haussinger D Giannaris T Shan M Moscovici M Voliotis D Bruix J Sharp Investigators Study Group Sorafenib in advanced hepatocellular carcinoma N Engl J Med 2008 359 378 390 10.1056/NEJMoa0708857 18650514

[B3] Mok TS Wu YL Thongprasert S Yang CH Chu DT Saijo N Sunpaweravong P Han B Margono B Ichinose Y Nishiwaki Y Ohe Y Yang JJ Chewaskulyong B Jiang H Duffield EL Watkins CL Armour AA Fukuoka M Gefitinib or carboplatin-paclitaxel in pulmonary adenocarcinoma N Engl J Med 2009 361 947 957 10.1056/NEJMoa0810699 19692680

[B4] Zhang J Yang PL Gray NS Targeting cancer with small molecule kinase inhibitors Nat Rev Cancer 2009 9 28 39 10.1038/nrc2559 19104514 PMC12406740

[B5] May R Riehl TE Hunt C Sureban SM Anant S Houchen CW Identification of a novel putative gastrointestinal stem cell and adenoma stem cell marker, doublecortin and CaM kinase-like-1, following radiation injury and in adenomatous polyposis coli/multiple intestinal neoplasia mice Stem Cells 2008 26 630 637 10.1634/stemcells.2007-0621 18055444

[B6] May R Sureban SM Lightfoot SA Hoskins AB Brackett DJ Postier RG Ramanujam R Rao CV Wyche JH Anant S Houchen CW Identification of a novel putative pancreatic stem/progenitor cell marker DCAMKL-1 in normal mouse pancreas Am J Physiol Gastrointest Liver Physiol 2010 299 G303 G310 10.1152/ajpgi.00146.2010 20522640 PMC2928534

[B7] Sureban SM May R Ramalingam S Subramaniam D Natarajan G Anant S Houchen CW Selective blockade of DCAMKL-1 results in tumor growth arrest by a Let-7a MicroRNA-dependent mechanism Gastroenterology 2009 137 649 659 659 e641-642 10.1053/j.gastro.2009.05.004 19445940 PMC2775069

[B8] Sureban SM May R Lightfoot SA Hoskins AB Lerner M Brackett DJ Postier RG Ramanujam R Mohammed A Rao CV Wyche JH Anant S Houchen CW DCAMKL-1 regulates epithelial-mesenchymal transition in human pancreatic cells through a miR-200a-dependent mechanism Cancer Res 2011 71 2328 2338 10.1158/0008-5472.CAN-10-2738 21285251 PMC3072762

[B9] Bailey JM Alsina J Rasheed ZA McAllister FM Fu YY Plentz R Zhang H Pasricha PJ Bardeesy N Matsui W Maitra A Leach SD DCLK1 marks a morphologically distinct subpopulation of cells with stem cell properties in pre-invasive pancreatic cancer Gastroenterology 2013 146 245 256 24096005 10.1053/j.gastro.2013.09.050 PMC3910427

[B10] Gerbe F van Es JH Makrini L Brulin B Mellitzer G Robine S Romagnolo B Shroyer NF Bourgaux JF Pignodel C Clevers H Jay P Distinct ATOH1 and Neurog3 requirements define tuft cells as a new secretory cell type in the intestinal epithelium J Cell Biol 2011 192 767 780 10.1083/jcb.201010127 21383077 PMC3051826

[B11] Nakanishi Y Seno H Fukuoka A Ueo T Yamaga Y Maruno T Nakanishi N Kanda K Komekado H Kawada M Isomura A Kawada K Sakai Y Yanagita M Kageyama R Kawaguchi Y Taketo MM Yonehara S Chiba T Dclk1 distinguishes between tumor and normal stem cells in the intestine Nat Genet 2012 45 98 103 10.1038/ng.2481 23202126

[B12] Verissimo CS Molenaar JJ Meerman J Puigvert JC Lamers F Koster J Danen EH van de Water B Versteeg R Fitzsimons CP Vreugdenhil E Silencing of the microtubule-associated proteins doublecortin-like and doublecortin-like kinase-long induces apoptosis in neuroblastoma cells Endocr Relat Cancer 2010 17 399 414 10.1677/ERC-09-0301 20228126

[B13] Verissimo CS Elands R Cheng S Saaltink DJ Ter Horst JP Alme MN Pont C van de Water B Havik B Fitzsimons CP Vreugdenhil E Silencing of Doublecortin-Like (DCL) results in decreased mitochondrial activity and delayed neuroblastoma tumor growth PLoS One 2013 8 e75752 10.1371/journal.pone.0075752 24086625 PMC3784435

[B14] Sureban SM May R Mondalek FG Qu D Ponnurangam S Pantazis P Anant S Ramanujam RP Houchen CW Nanoparticle-based delivery of siDCAMKL-1 increases microRNA-144 and inhibits colorectal cancer tumor growth via a Notch-1 dependent mechanism J Nanobiotechnol 2011 9 40 10.1186/1477-3155-9-40 PMC3200989 21929751

[B15] Sureban SM May R Qu D Weygant N Chandrakesan P Ali N Lightfoot SA Pantazis P Rao CV Postier RG Houchen CW DCLK1 Regulates Pluripotency and Angiogenic Factors via microRNA-Dependent Mechanisms in Pancreatic Cancer PLoS One 2013 8 e73940 10.1371/journal.pone.0073940 24040120 PMC3767662

[B16] Liu GH Qu J Suzuki K Nivet E Li M Montserrat N Yi F Xu X Ruiz S Zhang W Wagner U Kim A Ren B Li Y Goebl A Kim J Soligalla RD Dubova I Thompson J Yates J 3rd Esteban CR Sancho-Martinez I Izpisua Belmonte JC Progressive degeneration of human neural stem cells caused by pathogenic LRRK2 Nature 2012 491 603 607 10.1038/nature11557 23075850 PMC3504651

[B17] Deng X Dzamko N Prescott A Davies P Liu Q Yang Q Lee JD Patricelli MP Nomanbhoy TK Alessi DR Gray NS Characterization of a selective inhibitor of the Parkinson's disease kinase LRRK2 Nat Chem Biol 2011 7 203 205 10.1038/nchembio.538 21378983 PMC3287420

[B18] Thu KL Radulovich N Becker-Santos DD Pikor LA Pusic A Lockwood WW Lam WL Tsao MS SOX15 is a candidate tumor suppressor in pancreatic cancer with a potential role in Wnt/beta-catenin signaling Oncogene 2014 33 279 288 10.1038/onc.2012.595 23318427

[B19] Ross DT Scherf U Eisen MB Perou CM Rees C Spellman P Iyer V Jeffrey SS Van de Rijn M Waltham M Pergamenschikov A Lee JC Lashkari D Shalon D Myers TG Weinstein JN Botstein D Brown PO Systematic variation in gene expression patterns in human cancer cell lines Nat Genet 2000 24 227 235 10.1038/73432 10700174

[B20] Kantara C O'Connell M Sarkar S Moya S Ullrich R Singh P Curcumin Promotes Autophagic Survival of a Sub-Set of Colon Cancer Stem Cells, which are Ablated by DCLK1-siRNA Cancer Res 2014 10.1158/0008-5472.CAN-13-3536 PMC4013529 24626093

[B21] Ponnurangam S Mammen JM Ramalingam S He Z Zhang Y Umar S Subramaniam D Anant S Honokiol in combination with radiation targets notch signaling to inhibit colon cancer stem cells Mol Cancer Ther 2012 11 963 972 10.1158/1535-7163.MCT-11-0999 22319203 PMC3324630

[B22] Li L Bellows CF Doublecortin-like kinase 1 exhibits cancer stem cell-like characteristics in a human colon cancer cell line Chin J Cancer Res 2013 25 134 142 23592893 10.3978/j.issn.1000-9604.2013.03.02 PMC3626979

[B23] Duesberg P Stindl R Hehlmann R Origin of multidrug resistance in cells with and without multidrug resistance genes: chromosome reassortments catalyzed by aneuploidy Proc Natl Acad Sci U S A 2001 98 11283 11288 10.1073/pnas.201398998 11553793 PMC58721

[B24] Nishimoto S Nishida E MAPK signalling: ERK5 versus ERK1/2 EMBO Rep 2006 7 782 786 10.1038/sj.embor.7400755 16880823 PMC1525153

[B25] Watanabe K Akimoto Y Yugi K Uda S Chung J Nakamuta S Kaibuchi K Kuroda S Latent process genes for cell differentiation are common decoders of neurite extension length J Cell Sci 2012 125 2198 2211 10.1242/jcs.097709 22344266

[B26] Leushacke M Sporle R Bernemann C Brouwer-Lehmitz A Fritzmann J Theis M Buchholz F Herrmann BG Morkel M An RNA interference phenotypic screen identifies a role for FGF signals in colon cancer progression PLoS One 2011 6 e23381 10.1371/journal.pone.0023381 21853123 PMC3154943

[B27] Jurchott K Kuban RJ Krech T Bluthgen N Stein U Walther W Friese C Kielbasa SM Ungethum U Lund P Knosel T Kemmner W Morkel M Fritzmann J Schlag PM Birchmeier W Krueger T Sperling S Sers C Royer HD Herzel H Schafer R Identification of Y-box binding protein 1 as a core regulator of MEK/ERK pathway-dependent gene signatures in colorectal cancer cells PLoS Genet 2010 6 e1001231 10.1371/journal.pgen.1001231 21170361 PMC2996331

[B28] Itzkovitz S Lyubimova A Blat IC Maynard M van Es J Lees J Jacks T Clevers H van Oudenaarden A Single-molecule transcript counting of stem-cell markers in the mouse intestine Nat Cell Biol 2012 14 106 114 22119784 10.1038/ncb2384 PMC3292866

[B29] Van Landeghem L Santoro MA Krebs AE Mah AT Dehmer JJ Gracz AD Scull BP McNaughton K Magness ST Lund PK Activation of two distinct Sox9-EGFP-expressing intestinal stem cell populations during crypt regeneration after irradiation Am J Physiol Gastrointest Liver Physiol 2012 302 G1111 G1132 10.1152/ajpgi.00519.2011 22361729 PMC3362093

[B30] May R Qu D Weygant N Chandrakesan P Ali N Lightfoot SA Li L Sureban SM Houchen CW Dclk1 deletion in tuft cells results in impaired epithelial repair after radiation injury Stem Cells 2013 10.1002/stem.1566 PMC4603545 24123696

[B31] Schepers AG Snippert HJ Stange DE van den Born M van Es JH van de Wetering M Clevers H Lineage tracing reveals Lgr5+ stem cell activity in mouse intestinal adenomas Science 2012 337 730 735 10.1126/science.1224676 22855427

[B32] Proctor E Waghray M Lee CJ Heidt DG Yalamanchili M Li C Bednar F Simeone DM Bmi1 enhances tumorigenicity and cancer stem cell function in pancreatic adenocarcinoma PLoS One 2013 8 e55820 10.1371/journal.pone.0055820 23437065 PMC3577834

[B33] Ying H Kimmelman AC Lyssiotis CA Hua S Chu GC Fletcher-Sananikone E Locasale JW Son J Zhang H Coloff JL Yan H Wang W Chen S Viale A Zheng H Paik JH Lim C Guimaraes AR Martin ES Chang J Hezel AF Perry SR Hu J Gan B Xiao Y Asara JM Weissleder R Wang YA Chin L Cantley LC DePinho RA Oncogenic Kras maintains pancreatic tumors through regulation of anabolic glucose metabolism Cell 2012 149 656 670 10.1016/j.cell.2012.01.058 22541435 PMC3472002

[B34] Phipps AI Buchanan DD Makar KW Win AK Baron JA Lindor NM Potter JD Newcomb PA KRAS-mutation status in relation to colorectal cancer survival: the joint impact of correlated tumour markers Br J Cancer 2013 108 1757 1764 10.1038/bjc.2013.118 23511557 PMC3668469

[B35] Rhim AD Mirek ET Aiello NM Maitra A Bailey JM McAllister F Reichert M Beatty GL Rustgi AK Vonderheide RH Leach SD Stanger BZ EMT and dissemination precede pancreatic tumor formation Cell 2012 148 349 361 10.1016/j.cell.2011.11.025 22265420 PMC3266542

[B36] Chaffer CL Weinberg RA A perspective on cancer cell metastasis Science 2011 331 1559 1564 10.1126/science.1203543 21436443

[B37] Valenta T Hausmann G Basler K The many faces and functions of beta-catenin EMBO J 2012 31 2714 2736 10.1038/emboj.2012.150 22617422 PMC3380220

[B38] Sanchez-Tillo E de Barrios O Siles L Cuatrecasas M Castells A Postigo A beta-catenin/TCF4 complex induces the epithelial-to-mesenchymal transition (EMT)-activator ZEB1 to regulate tumor invasiveness Proc Natl Acad Sci U S A 2011 108 19204 19209 10.1073/pnas.1108977108 22080605 PMC3228467

[B39] Cancer Genome Atlas N Comprehensive molecular characterization of human colon and rectal cancer Nature 2012 487 330 337 10.1038/nature11252 22810696 PMC3401966

[B40] Shimomura S Nagamine T Nimura T Sueyoshi N Shigeri Y Kameshita I Expression, characterization, and gene knockdown of zebrafish doublecortin-like protein kinase Arch Biochem Biophys 2007 463 218 230 10.1016/j.abb.2007.03.036 17498644

[B41] Lin PT Gleeson JG Corbo JC Flanagan L Walsh CA DCAMKL1 encodes a protein kinase with homology to doublecortin that regulates microtubule polymerization J Neurosci 2000 20 9152 9161 11124993 10.1523/JNEUROSCI.20-24-09152.2000 PMC6773030

[B42] Nagamine T Shimomura S Sueyoshi N Kameshita I Influence of Ser/Pro-rich domain and kinase domain of double cortin-like protein kinase on microtubule-binding activity J Biochem 2011 149 619 627 10.1093/jb/mvr013 21278387

[B43] Davis MI Hunt JP Herrgard S Ciceri P Wodicka LM Pallares G Hocker M Treiber DK Zarrinkar PP Comprehensive analysis of kinase inhibitor selectivity Nat Biotechnol 2011 29 1046 1051 10.1038/nbt.1990 22037378

[B44] Wacker SA Houghtaling BR Elemento O Kapoor TM Using transcriptome sequencing to identify mechanisms of drug action and resistance Nat Chem Biol 2012 8 235 237 10.1038/nchembio.779 22327403 PMC3281560

[B45] Dhillon AS Hagan S Rath O Kolch W MAP kinase signalling pathways in cancer Oncogene 2007 26 3279 3290 10.1038/sj.onc.1210421 17496922

[B46] Kuribara M Jenks BG Dijkmans TF de Gouw D Ouwens DT Roubos EW Vreugdenhil E Scheenen WJ ERK-regulated double cortin-like kinase (DCLK)-short phosphorylation and nuclear translocation stimulate POMC gene expression in endocrine melanotrope cells Endocrinology 2011 152 2321 2329 10.1210/en.2011-0067 21447633

[B47] Miyamoto-Sato E Fujimori S Ishizaka M Hirai N Masuoka K Saito R Ozawa Y Hino K Washio T Tomita M Yamashita T Oshikubo T Akasaka H Sugiyama J Matsumoto Y Yanagawa H A comprehensive resource of interacting protein regions for refining human transcription factor networks PLoS One 2010 5 e9289 10.1371/journal.pone.0009289 20195357 PMC2827538

[B48] Pettersen EF Goddard TD Huang CC Couch GS Greenblatt DM Meng EC Ferrin TE UCSF Chimera–a visualization system for exploratory research and analysis J Comput Chem 2004 25 1605 1612 10.1002/jcc.20084 15264254

[B49] Yang Y Faraggi E Zhao H Zhou Y Improving protein fold recognition and template-based modeling by employing probabilistic-based matching between predicted one-dimensional structural properties of query and corresponding native properties of templates Bioinformatics 2011 27 2076 2082 10.1093/bioinformatics/btr350 21666270 PMC3137224

[B50] Schneidman-Duhovny D Inbar Y Polak V Shatsky M Halperin I Benyamini H Barzilai A Dror O Haspel N Nussinov R Wolfson HJ Taking geometry to its edge: fast unbound rigid (and hinge-bent) docking Proteins 2003 52 107 112 10.1002/prot.10397 12784375

[B51] Laskowski RA Swindells MB LigPlot+: multiple ligand-protein interaction diagrams for drug discovery J Chem Inf Model 2011 51 2778 2786 10.1021/ci200227u 21919503

[B52] Schwede T Kopp J Guex N Peitsch MC SWISS-MODEL: An automated protein homology-modeling server Nucleic Acids Res 2003 31 3381 3385 10.1093/nar/gkg520 12824332 PMC168927

[B53] Chang L-J Zaiss A-K Morgan J Lentiviral vectors preparation and use Gene Therapy Protocols, Volume 69 2002 New York: Springer 303 318 Methods in Molecular Medicine 10.1385/1-59259-141-8:303 11987785

[B54] Wang H Wang F Tao X Cheng H Ammonia-containing dimethyl sulfoxide: an improved solvent for the dissolution of formazan crystals in the 3-(4,5-dimethylthiazol-2-yl)-2,5-diphenyl tetrazolium bromide (MTT) assay Anal Biochem 2012 421 324 326 10.1016/j.ab.2011.10.043 22100715

[B55] Rouwette TP Kozicz T Olde Loohuis NF Gaszner B Vreugdenhil E Scheffer GJ Roubos EW Vissers KC Scheenen WJ Acute pain increases phosphorylation of DCLK-long in the Edinger-Westphal nucleus but not in the hypothalamic paraventricular nucleus of the rat J Pain 2010 11 930 940 10.1016/j.jpain.2009.12.017 20418180

