# Supplementary material for: Small molecule kinase inhibitor LRRK2-IN-1 demonstrates potent activity against colorectal and pancreatic cancer through inhibition of doublecortin-like kinase 1
Source: Mol Cancer. 2014 May 6;13:103. doi: 10.1186/1476-4598-13-103 (PMC4030036; doi:10.1186/1476-4598-13-103)
Supplement: Additional file 1: Table S1 — Primers sequence for genes analyzed by real-time RT-PCR. Figure S1. Cladogram of proteins closely related to DCLK1 as determined by ClustalΩ using the human kinome as input. Highlighting denotes solved structures (A) Anti- proliferative effect of LRRK2-IN-1 on colon and pancreatic cancer cell lines 48 h post-treatment (B). Figure S2. Two-dimensional plot of LRRK2-IN-1 and interacting residues as plotted by LigPlot and color-coded to agree with Figure 1E Red rays denote hydrophobic interactions and the green dashed line denotes 2.63A length hydrogen bonding between the ligand and Aspartate 226. Residue labeling agrees with the SwissProt entry for DCLK1 isoform 4 (A). Fluorescent images of cells stained with Calcein-AM (Live/Green) and Eth-D (Dead/Red) following LRRK2-IN-1 treatment (B). NCBI Geo data of DCLK1 gene expression in various pancreatic cancer cell lines compared to HPDE immortalized normal human pancreatic ductal epithelial cells (GSE40099) and comparison of DCLK1 gene expression in colon cancer cell lines from the NCI-60 cell panel (GDS 1761) (C) Exponential plots demonstrating strong associations between cell death and caspase activity and percentage of G2/M arrest (D-E) NCBI Geo data of DCLK1 gene expression in colon cancer cell lines treated with 10 μM of U0126 (F). [file 1476-4598-13-103-S1.pdf]

Supplementary Materials and Figures

Supplementary Table 1. Primer sequences for genes analyzed by real-time RT-PCR.

Gene ID	Forward (5'-3')	Reverse (5'-3')
**MYC**	CACACATCAGCACAACTACGCA	TTGACCCTCTTGGCAGGCAG
**DCLK1**	TAGCCAGCGCCATCAAATAC	ACCCAGCTTCAGTGATTTGC
**KRAS**	GACGATACAGCTAATTCAG	AGACAGGTTTCTCCATC
**NOTCH2**	TGGGCTACACTGGGAAAAAC	ACATAGGCACTGGGACTCTG
**NOTCH3**	TCACCATGCCGTAACGGG	TCGGTGTCCTGGACAGTCG
**NOTCH4**	GGCCTCGGACTCAGTCAGC	CAACTCCATCCTCATCAACTTCTG
**REG4**	CCAGAAGCATGCGGCTGCTC	GTGGTAAAACCATCCAGGAGCAC
**TWIST1**	GCCGGAGACCTAGATGTCATTG	AGTTATCCAGCTCCAGAGTCTC
**WNT1**	TAAGCAGGTTCGTGGAGGAG	GGTTTCTGCTACGCTGCTG
**BCL2**	TGGATCCAGGATAACGGAGG	CAAACAGAGGTCGCATGCTG
**BCL2L1**	TCTACGGGAACAATGCAGCA	AGGAACCAGCGGTTGAAGC
**MCL1**	AAGGACACAAAGCCAATGGG	ACTCCACAAACCCATCCTTGG
**LIN28**	GATGTATTTGTACACCAA	TACCCGTATTGACTCAAGGCC
**NANOG**	ACCAGAACTGTGTTCTCTTCCACC	CCATTGCTATTCTTCGGCCAGTTG
**SOX2**	CGAGATAAACATGGCAATCAAAAT	AATTCAGCAAGAAGCCTCTCCTT
**BMI1**	GAAAGTTTCTCAGAAGTAAAATGG	TGTAGGCAATATCCATTAGTGTAT
**LGR5**	TTAGAGACATGGGACAAATGCCAC	AACAGTCCTGTGACTCAACTCAACTCAAG
**SNAI1**	GCCATGTCCGGACCCACACTG	GGCAGGGGCAGGTATGGAGA
**ZEB1**	AAGAATTCACAGTGGAGAGAGAAGCCA	CGTTTCTTGCAGTTTGGGCATT
**ZEB2**	AGCCGATCATGGCGGATGGC	TTCCTCCTGCTGGGATTGGCTTG
**CHD1**	CCTCCCATCAGCTGCCC	GTGATGCTGTAGAAAACCTT
**ACTB**	GCTGATCCACATCTGCTGG	ATCATTGCTCCTCCTGAGCG

Supplementary Figure Legends

Supplementary Figure 1 (S1). Cladogram of proteins closely related to DCLK1 as determined by ClustalΩ using the human kinome as input. Highlighting denotes solved structures (**A**). Anti-proliferative effect of LRRK2-IN-1 on colon and pancreatic cancer cell lines 48 h post-treatment (**B**).

Supplementary Figure 2 (S2). Two-dimensional plot of LRRK2-IN-1 and interacting residues as plotted by LigPlot and color-coded to agree with **Fig 1E**. Red rays denote hydrophobic interactions and the green dashed line denotes 2.63Å length hydrogen bonding between the ligand and Aspartate 226. Residue labeling agrees with the SwissProt entry for DCLK1 isoform 4 (**A**). Fluorescent images of cells stained with Calcein-AM (Live/Green) and Eth-D (Dead/Red) following LRRK2-IN-1 treatment (**B**). NCBI Geo data of DCLK1 gene expression in various pancreatic cancer cell lines compared to HPDE immortalized normal human pancreatic ductal epithelial cells (GSE40099) and comparison of DCLK1 gene expression in colon cancer cell lines from the NCI-60 cell panel (GDS1761) (**C**). Exponential plots demonstrating strong associations between cell death and caspase activity and percentage of G2/M arrest (**D-E**). NCBI Geo data of DCLK1 gene expression in colon cancer cell lines treated with 10 µM of U0126 (**F**).

A.

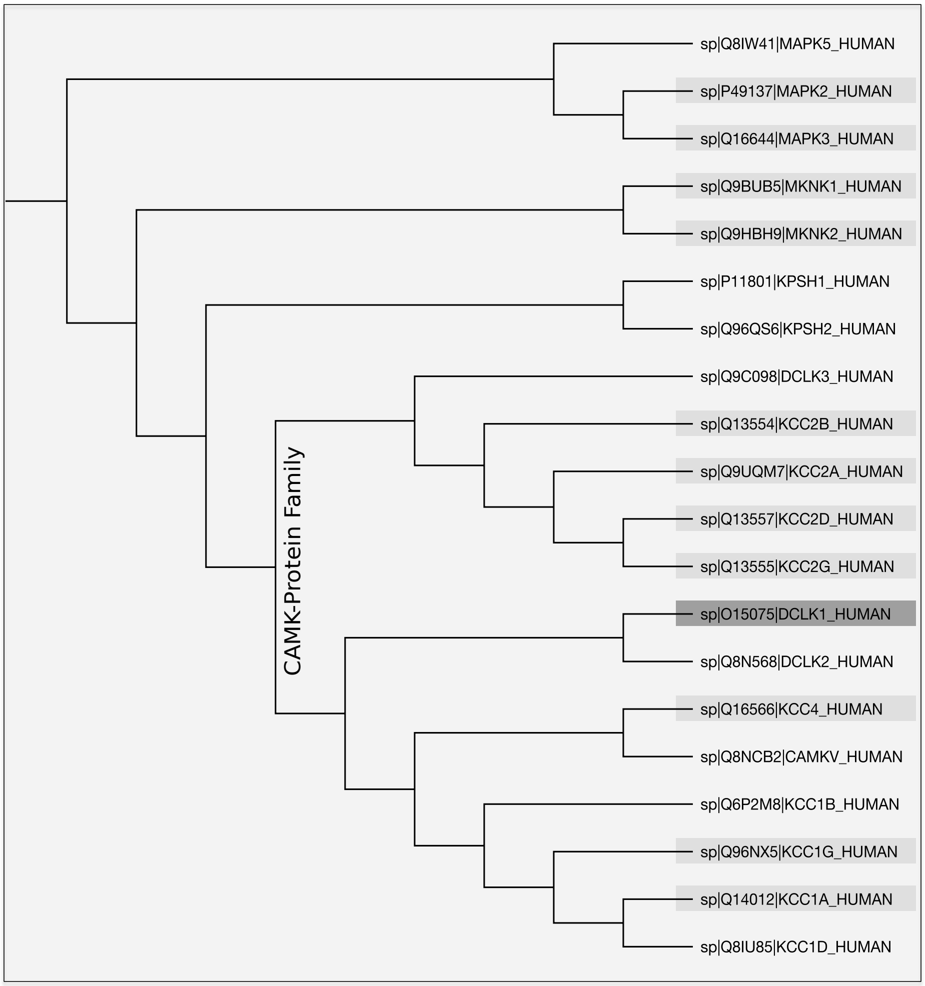

B.

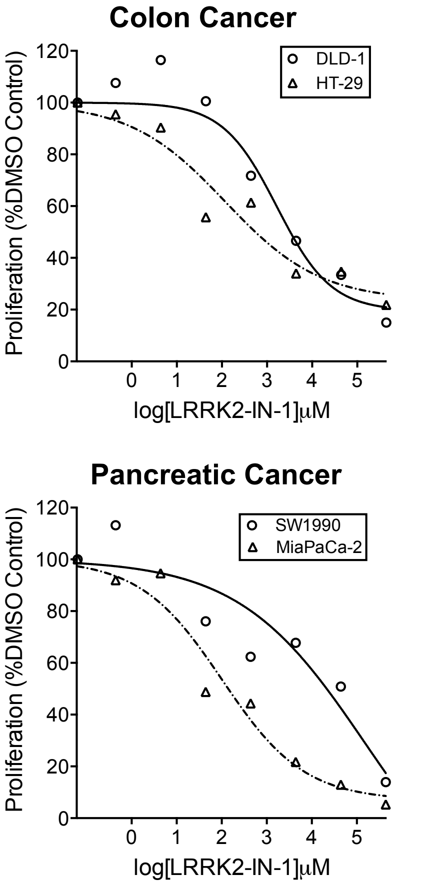

**Cell Line**	**IC50 (μM)**	**R^2**
DLD-1	3.219	0.93
HT-29	2.073	0.89
MiaPaCa-2	2.010	0.91
SW1990	5.191	0.73

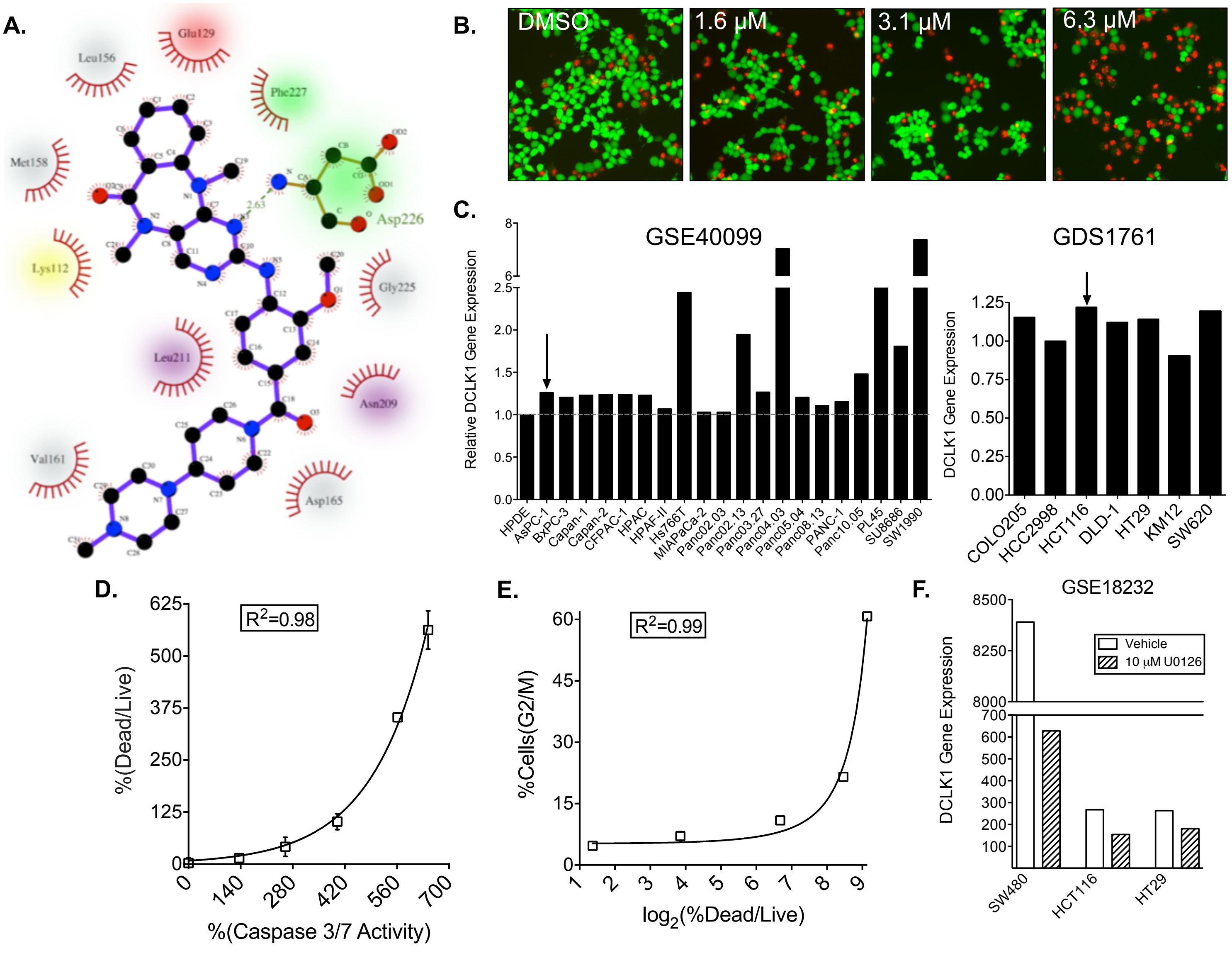
A.
Glu129
Leu156
Phe227
Met158
Asp226
Lys112
Gly225
Leu211
Asn209
Val161
Asp165
2.63
B.
DMSO
1.6 μM
3.1 μM
6.3 μM
C.
GSE40099
Relative DCLK1 Gene Expression
HPDE
AsPC-1
BxPC-3
Capan-1
Capan-2
CFPAC-1
HPAC
HPAF-II
Hs766T
MIAPaCa-2
Panc02.03
Panc02.13
Panc03.27
Panc04.03
Panc05.04
Panc08.13
PANC-1
Panc10.05
PL45
SU8686
SW1990
GDS1761
DCLK1 Gene Expression
COLO205
HCC2998
HCT116
DLD-1
HT29
KM12
SW620
D.
R^2=0.98
%(Dead/Live)
%(Caspase 3/7 Activity)
E.
R^2=0.99
%Cells(G2/M)
$\log_2$(%Dead/Live)
F.
GSE18232
DCLK1 Gene Expression
Vehicle
10 μM U0126
SW480
HCT116
HT29
